# ORAL CONCURRENT SESSION 9: Medical Complications

**DOI:** 10.1002/pmf2.12021

**Published:** 2025-01-05

Abstracts 95 – 104

SATURDAY

February 1, 2025

8:00 AM – 10:30 AM

Aurora Ballroom CD

MODERATORS

Washington Clark Hill, MD

Meredith Rochon, MD

